# A novel mass spectrometric strategy “BEMAP” reveals Extensive O-linked protein glycosylation in Enterotoxigenic *Escherichia coli*

**DOI:** 10.1038/srep32016

**Published:** 2016-08-26

**Authors:** Anders Boysen, Giuseppe Palmisano, Thøger Jensen Krogh, Iain G. Duggin, Martin R. Larsen, Jakob Møller-Jensen

**Affiliations:** 1 Department of Biochemistry and Molecular Biology, University of Southern Denmark, Campusvej 55, 5230 Odense M, Denmark; 2 The ithree institute, University of Technology Sydney, PO Box 123, Broadway NSW 2007, Australia; 3 GlycoProteomics laboratory, Department of Parasitology, University of Sao Paulo, Brazil

## Abstract

The attachment of sugars to proteins via side-chain oxygen atoms (O-linked glycosylation) is seen in all three domains of life. However, a lack of widely-applicable analytical tools has restricted the study of this process, particularly in bacteria. In *E. coli*, only four O-linked glycoproteins have previously been characterized. Here we present a glycoproteomics technique, termed BEMAP, which is based on the beta-elimination of O-linked glycans followed by Michael-addition of a phosphonic acid derivative, and subsequent titanium dioxide enrichment. This strategy allows site-specific mass-spectrometric identification of proteins with O-linked glycan modifications in a complex biological sample. Using BEMAP we identified cell surface-associated and membrane vesicle glycoproteins from Enterotoxigenic *E. coli* (ETEC) and non-pathogenic *E. coli* K-12. We identified 618 glycosylated Serine and Threonine residues mapping to 140 proteins in ETEC, including several known virulence factors, and 34 in *E. coli* K-12. The two strains had 32 glycoproteins in common. Remarkably, the majority of the ETEC glycoproteins were conserved in both strains but nevertheless were only glycosylated in the pathogen. Therefore, bacterial O-linked glycosylation is much more extensive than previously thought, and is especially important to the pathogen.

Enterotoxigenic *Escherichia coli* (ETEC) is the major source of *E. coli* mediated diarrhoea in humans and livestock^1^. ETEC infections cause more than 280 million annual episodes of diarrhoea resulting in mortality numbers exceeding 550,000 deaths of children under the age of five years^2^. The significant negative health- and socio-economic impact of ETEC infection manifests itself mainly in the undeveloped nations with poor sanitation and inadequate supplies of clean water^3^. ETEC strains are a diverse group of pathogens defined by their ability to colonize the small intestine and secrete heat-labile and/or heat stable enterotoxins^4^. Their pathogenicity is further attributed to the presence of virulence genes on mobile genetic elements, including a number of plasmids and chromosomal pathogenicity islands^5^.

Much attention has been devoted to the understanding of how ETEC and other mucosa-associated pathogens interact with host tissue during infection^6^. A number of studies have revealed that bacterial protein glycosylation plays an important role in mediating adhesion, colonization and invasion of host tissue^7^,^8^,^9^,^10^,^11^,^12^. However, up until now, the known protein glycosylation repertoire of *E. coli* was limited to just four proteins, all of which are surface-exposed adhesins with functions in bacterial pathogenesis^13^,^14^,^15^,^16^.

While the intimate coupling between protein glycosylation and bacterial pathophysiology has become apparent, the discovery of novel glycoproteins implicated in virulence is advancing slowly^6^,^17^,^18^. This is attributed to the inherent challenges associated with glycoproteomics. The analytical tools developed for enrichment of eukaryotic O- and N-linked glycopeptides rely on a limited set of defined physiochemical properties, *e.g.* glycan hydrophilicity or specific lectin recognition, which are relatively rare in bacteria^19^,^20^. Discovery and characterization of glycoproteins is further complicated by heterogeneous glycosylation, low abundance, poor ionization of peptides modified with carbohydrates compared to the non-modified counterpart^21^ and lack of specific enzymes to remove the heterogeneous glycan structure prior to mass spectrometric (MS) analysis^22^.

Mapping of O-linked glycan moieties has also proven to be challenging owing to the diverse nature of carbohydrate structures available for protein modification in bacteria^23^. Although methods such as periodic acid/hydrazide glycan labelling and metabolic oligosaccharide engineering (MOE) have identified glycoproteins in a range of bacteria, these techniques present limitations in the form of low specificity for glycosylated proteins and dependence on sugar uptake and integration into bacterial glycoproteins, respectively^17^,^24^. Recently, the diversity within the O-linked protein glycosylation systems of Acinetobacter species were described using Hydrophilic Interaction LIquid Chromatography (HILIC) glycopeptide enrichment in combination with ETD HCD and CID fragmentation in the MS instrument^25^.

Here we describe a novel mass spectrometry-based technique, termed BEMAP, which can be employed to map O-linked glycoproteins from theoretically any biological source. BEMAP is an extension of a method, which was originally described for phosphorylated peptides enrichment analysis of phosphorylated proteins as a tool for probing the phosphoproteome^26^ and later for O-GlcNAcylated peptides^27^. In this strategy the phosphate or O-GlcNAc moieties are replaced by an affinity tag, commonly a biotin group, using base-catalyzed β-elimination followed by Michael-addition of the affinity tag.

The BEMAP reaction efficiently substitutes O-linked carbohydrate moieties with a 2-Aminoethyl phosphonic acid (AEP) group, which can be selectively isolated based on its affinity for titanium dioxide, as previously shown for phosphorylated peptides^28^. Here we employed the BEMAP strategy to map 618 novel protein O-glycosylation sites in the ETEC strain H10407 and the non-pathogenic *E. coli* K-12. The far majority of the sites were identified in the pathogenic *E. coli* strain. These results highlight protein O-glycosylation in bacteria as an abundant, yet largely unexplored, post-translational protein modification associated with cellular core processes and pathophysiology. Bacterial O-glycoproteins potentially constitute an important reservoir of novel therapeutic targets, biomarkers and vaccine candidates.

## Results and Discussion

### BEMAP enables selective and efficient enrichment of O-linked glycopeptides

The intimate coupling between bacterial protein glycosylation and pathophysiology found in several species^6^ prompted us to identify novel glycosylated proteins in ETEC strain H10407. ETEC modifies proteins with heptose and potentially N-acetylglucosamine (GlcNAc) monosaccharides^15^,^16^. Due to the lack of suitable methods to investigate the O-linked glycoproteome in bacteria, we developed a mass spectrometry-based technique for unbiased identification of O-linked protein glycosylation sites on a proteome scale.

Selective enrichment of phosphopeptides and O-GlcNAc modified glycopeptides was previously demonstrated by use of phosphate tag, which has a high affinity for TiO_2_^28^,^29^,^30^. Our method, termed BEMAP, relies on β-elimination of O-linked carbohydrate modifications, Michael addition of 2-Aminoethyl phosphonic acid (AEP), and then subsequent titanum dioxide (TiO_2_) enrichment of the resulting phosphonate peptides. Thus, BEMAP combines an established *in vitro* chemical modification with a highly selective enrichment protocol^31^. The reactions take place in a single sample without the need for intermediate purification steps (see Experimental procedures section).

**Figure 1:**
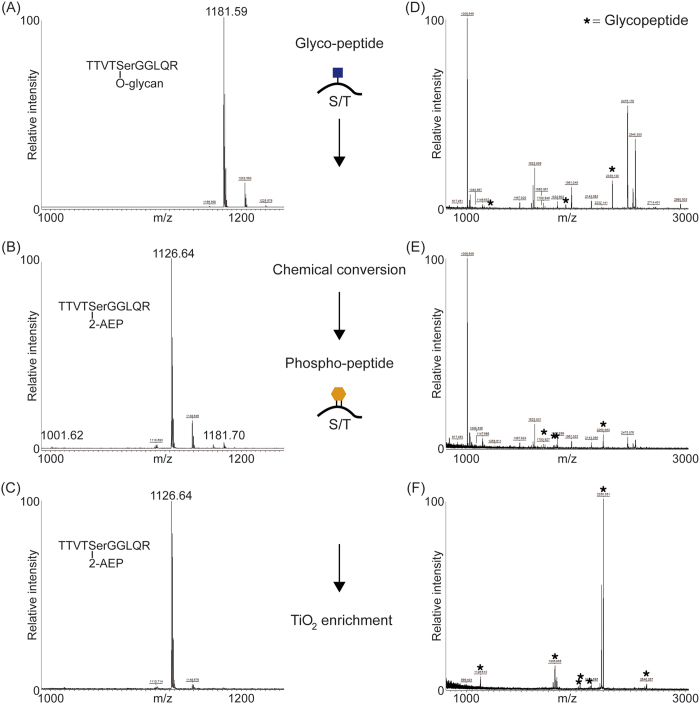
β-Elimination of glycan moiety and replacement with 2-AEP through Michael addition chemistry. (**A**) MALDI MS spectrum of TTVTSGGLQR (m/z = 1181.59 Da) synthetic O-linked glycopeptide. (**B**) The BEMAP reaction efficiently replaces the carbohydrate moiety with the 2-AEP molecule and produces a phosphopeptide with the mass of 1126.64 Da. Minor traces of beta-eliminated as well as intact peptide can be observed (m/z = 1001.62 Da and 1181.59, receptively). (**C**) The AEP modified peptide is selectively enriched with TiO_2_ as both the glycopeptide and the beta-eliminated peptide is absent in the MALDI MS spectrum. (**D**) MALDI MS peptide mass fingerprint of Tryptic digest of heptosylated protein Ag43. Ag43 can be digested into a mixture of heptosylated as well as unmodified peptides. Peptides marked with an asterisk indicate heptosylation. (**E**) BEMAP converts heptosylated peptides into phosphopeptides; modified peptides are indicated. (**F**) Specific TiO_2_ enrichment of phosphopeptides.

The BEMAP method was first established using a synthetic O-linked mannosylated peptide as a model compound. As shown in Fig. 1A,B, MALDI MS demonstrated that BEMAP efficiently replaced the carbohydrate moiety of the synthetic peptide (m/z = 1181.59 Da) with the AEP group and thus introduced a phosphonate peptide (m/z = 1126.64 Da). The overall efficiency of substitution exceeded 95% (Fig. 1B) without the formation of any visual degradation products. The AEP-modified peptide was then efficiently enriched using TiO_2_ affinity chromatography; both the intact glycopeptide and the β-eliminated peptide (1001.62 Da) were absent in the MALDI MS spectrum after enrichment (Fig. 1C). We found that BEMAP converted O-linked glycopeptides into phosphonate peptides in a time dependent fashion and that maximum conversion had occurred after 195 minutes (Supplementary Fig. S1A–E).

We analysed the gas-phase fragmentation properties of the converted O-linked glycopeptide. As shown in Supplementary Fig. S2, the exchange of a carbohydrate moiety with AEP has several advantages. The AEP addition substitutes a labile glycoside bond with a stronger covalent C-N bond, which greatly improved mapping of O-glycosylated amino acid residues by high-energy C-trap dissociation (HCD) fragmentation (Supplementary Fig. S2). Moreover, the AEP group yielded two characteristic reporter ions during HCD fragmentation (m/z = 126.03 Da and m/z = 138.03 Da), which are very useful for glycopeptide identification and validation in complex MS/MS spectra. It should be noted that the AEP molecule has a phosphonate functional group, which is stable under CID and HCD fragmentation conditions in contrast to a phosphate group, which is labile under these conditions. This allows unambiguous assignment of the modified amino acid residues and avoids false positives in site localization assignment. In addition, HCD fragmentation yielded higher peptide sequence coverage and allows one to monitor the two reporter ions (Supplementary Fig. S2). Due to that, HCD fragmentation was used in other experiments. Of importance, even though the synthetic glycopeptide contains three threonine residues, only the O-linked serine amino acid was converted into a phosphonate peptide by the BEMAP chemistry. This implies a specific chemical reactivity towards O-linked glycans compared to unmodified serine/threonine residues. Next, we applied BEMAP to a purified heptosylated protein: Ag43 from *E. coli*^32^. As seen in Fig. 1D, in-gel trypsin digestion of the glycosylated protein yielded heptosylated and unmodified peptides. Heptosylated peptides marked by an asterisk are suppressed by the non-modified peptides. However, the BEMAP strategy enriched the three heptosylated peptides present in Fig. 1D as well as four additional glycopeptides initially undetectable by MALDI MS (Fig. 1E,F). It is concluded that BEMAP is a specific and sensitive method for identification of formerly O-linked glycosylated peptides.

### Enzymatic dephosphorylation treatment required for large-scale glycoproteome analyses

Both pathogenic and commensal *E. coli* phosphorylates a subset of its proteome^33^,^34^,^35^,^36^. As the BEMAP chemistry relies on β-elimination and Michael addition, which previously has been shown to react with serine and threonine phosphorylation^26^ we therefore considered phosphoprotein as a potentially confounding factor in identification of the genuine O-glycoproteome. Moreover, the TiO_2_ enrichment step included in the workflow could potentially affinity-purify phosphopeptides not converted into 2-AEP peptides and suppress the ionization of 2-AEP modified O-glycopeptides. In order to evaluate the extent of such false identification, we first assessed how efficiently BEMAP converts phosphorylated serine and threonine residues into 2-AEP modified peptides. Using the experimental conditions described above, we studied the multiply phosphorylated protein Fetuin using MALDI MS. TiO_2_ enriched phosphopeptides from in-solution digested Fetuin were isolated and then analysed by MALDI MS (Supplementary Fig. S3A)^28^. Assuming similar ionization efficiency of the phospho- and AEP-modified peptides by MALDI, we observe that treating the phosphopeptides with the BEMAP chemistry, results in less than 38% conversion of the mono-phosphopeptide to the corresponding phosphonate peptide. The di-phosphorylated peptide is converted to an even lesser extent. Of peptides containing two possible substitution sites, only 36% were converted on a single site. 83% of the tri-phosphorylated peptide were substituted with a single AEP modification; double- and triple substitutions were not observed (Supplementary Fig. S3B). The BEMAP conversion rate is similar to the one observed by Wells *et al*.^27^ but indicates that the chemistry could exchange serine and threonine phosphorylations with the 2-AEP tag.

We then performed an experiment in order to estimate the number of false positive identifications which could be expected in an outer membrane O-glycoproteome BEMAP analysis. In three biological experiments, proteins associated with the outer membrane were sequentially isolated^37^ and digested with trypsin. The peptides were treated either with or without alkaline phosphatase (AP) prior to phosphopeptide enrichment and LC-MS/MS analysis^28^. When analysing the samples not treated with AP prior to phosphopeptide enrichment and mass spectrometry, we identified 227 phosphorylated S/T/Y residues which could be assigned to 99 proteins (Supplementary Fig. S4A,B and Supplementary Table S1). In the parallel experiment, which included an AP sample treatment, we mapped 51 phosphorylation sites to 31 proteins. When comparing the two datasets, 23 phosphorylation sites were shared between 13 proteins, see Supplementary Fig. S4A,B. These results show that at the protein level, AP sample treatment significantly reduces the number of identified phosphoproteins as expected.

Taking into account, that only proteins associated with the outer membrane were sampled, we have identified a high number of phosphopeptides when comparing to the published in-depth phosphoproteome studies carried out in commensal MG1655 *E. coli*^33^,^35^,^36^. In the MG1655 analyses, the serine and threonine phosphorylation represented approximately 70% and 20% of all modifications, respectively. Less than 10% were phosphorylated tyrosine residues. We observe a similar ratio between pS, pT and pY. Although tyrosine phosphorylation is relatively rare, a phosphotyrosine immunoaffinity based pulldown experiment identified 512 modified residues in both commensal and pathogenic enterohemorrhagic *E. coli* (EHEC)^34^. In our analysis, S/T phosphopeptides are potentially a source for overestimating the O-glycoproteome. However, a low BEMAP chemistry conversion rate of phosphorylated S/T residues in combination with AP treatment prior to a BEMAP O-glycoproteome analysis removes the majority of the false positive identifications.

### Characterization of the *E. coli* outer membrane O-glycoproteome

**Figure 2:**
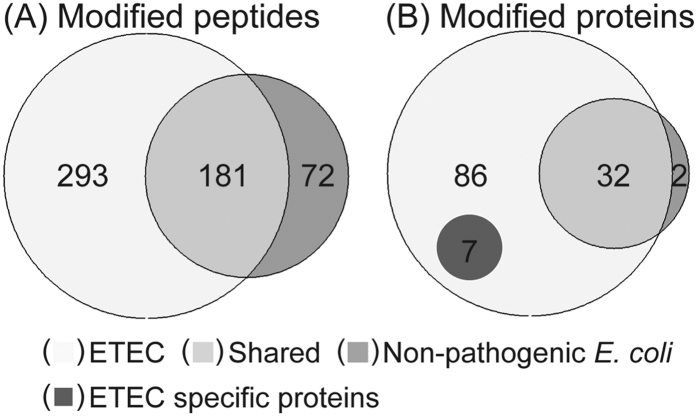
Comparison of quantitative differences between ETEC and commensal *E. coli* O-linked glycosylated proteins associated with the outer membrane. (**A**) Proportional numerical representation of identified glycopeptides in pathogenic ETEC and commensal *E. coli* strain. Intersecting circles indicate glycopeptides expressed in both strains. Color code: 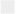 Unique to ETEC; 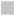 Glycopeptides identified in both strains; 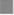 Unique to non-pathogenic *E. coli*. (**B**) Proportional numerical representation of identified glycoproteins in pathogenic and commensal strain. Intersecting circles indicate glycoproteins expressed in both strains. Color code: 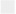 Unique to ETEC; 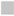 Glycopeptides identified in both strains; 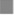 Unique to commensal *E. coli*; 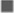 Proteins encoded by ETEC only. The list of unique modified sites was extracted and compiled using our in house developed software tool, see methods and materials.

The four known *E. coli* glycoproteins AIDA-1, Antigen 43 (Ag43), TibA and EtpA are all associated with the outer membrane, and we reasoned that additional glycoproteins involved in interacting with host cells could also be associated with the surface. We therefore sought to identify additional O-glycoproteins from the outer membrane of the ETEC strain H10407 using BEMAP. Interestingly, the closely related commensal *E. coli* K-12 strain MG1655 carries the potential for protein glycosylation at the genetic level^14^ and were thus included in the analysis as a non-pathogenic reference. If protein O-glycosylation could be identified in both *E. coli* strains, a direct comparison of the two related organisms sampled under identical conditions would potentially highlight important differences and similarities useful for predicting novel O-glycosylated therapeutic targets in ETEC. The outer membrane protein fractions of H10407 and MG1655 were isolated using exactly the same outer membrane- and TiO_2_ enrichment protocol as for phosphopeptide purification and subjected to BEMAP analysis for site specific identification of O-linked glycosylation sites. This identified a total of 547 glycosylated residues (Fig. 2A) which could be assigned to 127 proteins (Fig. 2B). A total of 125 glycosylated proteins were identified in the ETEC outer membrane, whereas 34 glycoproteins were found in the commensal strain (Fig. 2B). When using a sequence alignment with a 60% identity and 80% similarity cut-off threshold at the protein level, 32 proteins were shared between the two strains; leaving just 2 proteins only found modified by the non-pathogen, see Supplementary Table S2. Out of the 93 glycoproteins specifically identified in the ETEC sample only seven proteins were uniquely expressed in the pathogen (Fig. 2B), implying that ETEC O-glycosylates its protein to a much higher extent than to the non-pathogen, just as tyrosine phosphorylation has been proposed to be in EHEC^34^. This indicates that post-translational modifications (PTMs) could play a role in the pathophysiology of *E. coli*. Interestingly, some of these proteins, *e.g.* adhesin TibA autotransporter, colonization factor CfaB, pesticin/yersiniabactin TonB-dependent receptor and Yersiniabactin siderophore biosynthetic protein are associated with various aspects of pathogenesis such as host cell adhesion and iron acquisition (bold highlighted proteins in Supplementary Table S2)^4^,^38^. The adhesin TibA autotransporter has previously been described to be associated with the outer membrane^39^. The identification of TibA in our analysis validates the BEMAP enrichment (Supplementary Fig. S5A,B).

**Figure 3:**
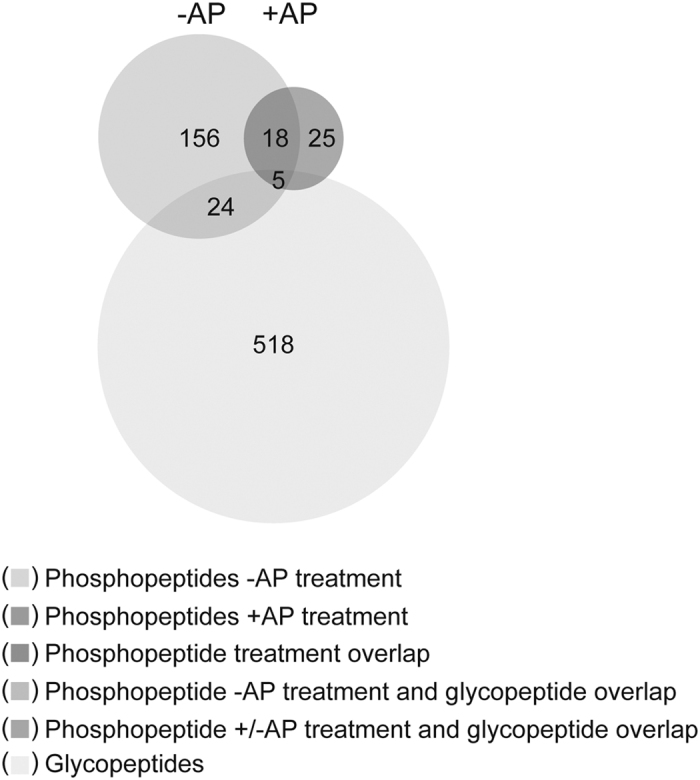
O-linked S/T phosphorylation and O-linked protein glycosylation are two distinct groups of post translational modifications in *E. coli*. Proteins associated with the outer membrane were isolated and treated either with or without Alkaline Phosphatase (AP) enzyme prior to O-linked phosphospeptide enrichment using TiO_2_ and LC MS/MS analysis. Proportional numerical representation was used to show identified S/T phosphorylated residues. Intersecting circles indicate phosphopeptides identified under the two different experimental conditions. Proportional numerical representation and intersecting circles is used to compare the overlap between phosphorylated and glycosylated residues expressed in both strains. Color code: 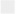 Number of phosphopeptides identified without AP treatment; 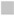 Number of phosphopeptides identified with AP treatment; 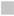 Number of phosphopeptide identified with or without AP treatment; 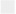 Number of phosphopeptides identified without AP treatment which also can be glycosylated; 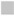 Number of phosphopeptide identified with or without AP treatment which also can be glycosylated.

Knowing that a sub-fraction of the identified O-glycopeptides could potentially be phosphopeptides (see Supplementary Table S1 and Table S2) we compared the list of O-linked glycopeptides to the list of phosphorylated serine- and threonine-phosphopeptides previously identified in the sample (Supplementary Table S3). As shown in Fig. 3, omitting AP treatment prior to phosphopeptide enrichment and LC-MS/MS analysis revealed that only 24 peptides of the 546 identified O-glycopeptides were shared between the two data sets. These 24 peptides could be assigned to nine proteins (Supplementary Table S3). Only 5 phosphorylated sites overlapped between the BEMAP enriched- and the phosphopeptide enriched samples following AP treatment (Fig. 3). We propose that these five particular sites are AP treatment resistant. We conclude that AP treatment can reduce the number of false positive identifications and that our BEMAP workflow identifies O-glycosylated peptides with high selectivity. Of note, less than 5% of the modified sites (29 out of 715 sites; Fig. 3) are overlapping, suggesting that protein phosphorylation and O-glycosylation occurs with different specificity. Furthermore, glycosylation appears to be a more abundant than phosphorylation, at least in the outer membrane fraction.

**Figure 4:**
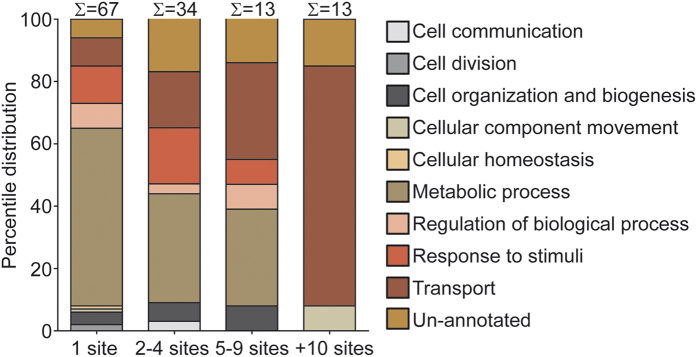
Characterization of ETEC O-linked outer membrane glycoproteome. The predicted biological processes of the outer membrane glycoproteome changes with an increasing number of glycosylations/protein. Absolute number of proteins within each group is shown above each column. Percentile distribution of each GO term is displayed. Color code: 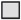 Cell communication; 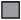 Cell division; 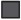 Cell organization and biogenesis; 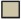 Cellular component movement; 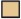 Cellular homeostasis; 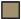 Metabolic process; 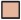 Regulation of biological process; 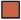 Response to stimulus; 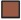 Transport; 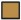 Unannotated.

In order to determine if protein O-glycosylation could be associated with specific cellular functions, the outer membrane associated glycoproteins were clustered into four groups according to the number of O-linked glycosylation sites. We additionally categorized the glycoproteins according to their gene ontology (GO) annotation^40^. As shown in Fig. 4, the clustering revealed that 101 of the 127 proteins had a limited number of O-glycosylated residues (1–4 sites).Twenty-six glycoproteins were more extensively modified, showing more than four distinct O-glycosylation sites each. Roughly 20% of the proteins in all four groups were of unknown function. Approximately half of all the proteins that carried up to four glycosylation sites were predicted to be metabolic enzymes whereas more than 70% of all proteins with more than 10 glycosylation sites were categorized as outer membrane transporters. Nine of theses transporters, CirA, FepA, FhuA, OmpC, OmpF, OmpA, Peptidoglycan-associated lipoprotein, OmpT and TolC are involved in acquisition of iron, hydrophilic solutes, ions as well as peptides. Since the majority of these have previously been crystallized (see Supplementary Table S2) we next investigated if the O-glycosylation sites were randomly distributed within the protein structure or if they clustered into particular spatial regions, which could be of biological importance. Therefore, we visualized the structural position of the O-glycosylation sites by highlighting the sugar-modified residues within crystal structures (Supplementary Fig. S6A-5I). Generally, the O-glycosylation sites were located in either unstructured regions on the protein exterior or positioned within the barrel pore. The spatial arrangement of the glycosylated residues suggest that the hydrophilic environment created by the monosaccharide is an integral function of the *E. coli* outer membrane transporters. It is striking that the carbohydrate modifications have gone by unnoticed in all published crystal structure we examined. We speculate that crystals used for X-ray diffraction require large amounts protein, which is usually obtained by overexpression in a non-native host, which may not necessarily express the cognate glycosyltranferases required for glycosylation or other PTMs. It is also possible that ectopic protein expression might deplete the host cell for nucleotide-activated sugar that is funneled into the glycosylation pathway thus rendering the protein un-modified.

### Identification of glycoproteins in ETEC membrane vesicles

Both pathogenic and commensal *E. coli* produce membrane vesicles (MVs) containing proteins^41^. However, when comparing the relative protein composition of MVs released by ETEC and MG1655, it has been shown that vesicles produced by the pathogen functions as a vessel for export of LT toxin and bacterial virulence factors targeting the host mucosal layer^42^,^43^. We isolated ETEC MVs and performed a BEMAP analysis in order to examine whether any of these proteins could be glycosylated. Using BEMAP, we identified 133 glycosylated residues which could be assigned to 22 proteins (Supplementary Table S4). Out of the 22 proteins, the known glycoproteins EtpA and TibA as well as Flagellin, EatA, YghJ, CfaB and CexE are characterized as ETEC virulence factors. The MVs also contained a number of proteins with putative unknown functions. The exact role of glycoproteins in pathogenesis remains to be determined but approximately 1/6 of all proteins identified in ETEC vesicles appear to be glycosylated^43^.

**Figure 5:**
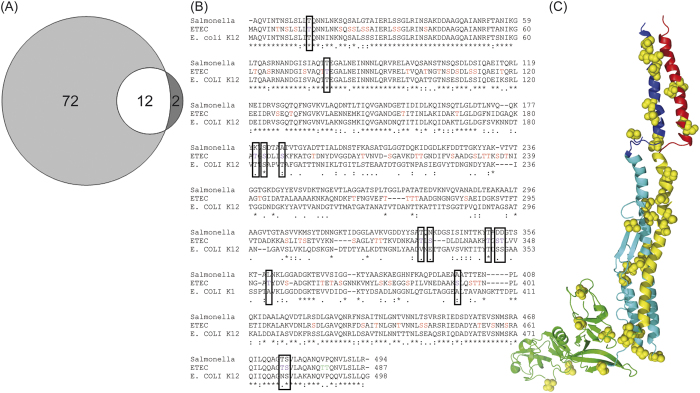
ETEC FliC protein glycosylation mapping. (**A**) Proportional numerical representation of identified Heptosylated peptides using either BEMAP or an orthogonal experimental approach. (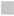) Number of phosphopeptides identified BEMAP; (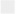) Number of phosphopeptides identified in both approaches; (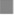)Number of phosphopeptide using an orthogonal approach (**B**) Clustal Omega W sequence alignment of *Salmonella typhimurium,* ETEC H10407 and *E. coli* K-12 MG16556 showing extent of sequence similarity. Boxed purple S and T amino acids indicate Heptosylated peptides identified using either BEMAP or an orthogonal experimental approach. Green colored S and T residues highlight glycosylation only identified using an orthogonal experimental workflow. Red colored S and T residues highlight ETEC glycosylation identified using BEMAP. The symbols *, and indicate sequence identity, sequence similarity among the three strains. (**C**) Visualization of ETEC FliC protein glycosylation using *Salmonella typhimurium* as template^71^. Glycosylated residues are shown as yellow spheres.

We observed that a number of different glycopeptides identified in both the outer membrane fraction and in MVs could be assigned to the same protein. Thus, we combined the two glycopeptide lists into a non-redundant dataset showing the full extent of protein O-glycosylation of proteins associated with the outer membrane in ETEC and commensal MG1655 (Supplementary Table S5). Quite remarkably, the combined list revealed that 84 H10407 FliC Ser/Thr residues out of 100 possible were modified. This extent of modification surpasses any reported number of O-linked glycosylation sites on a single protein in bacteria^6^,^44^. FliC (flagellin), the major structural component of the flagellum, is conserved in the two related *E. coli* strains. However, the number of glycosylated FliC residues in the two organisms is strikingly different. FliC is only glycosylated in ETEC (see Supplementary Table S2). Previous studies have shown that the flagellum is required for efficient adherence to the intestinal epithelium^45^ and our findings group ETEC with the mucosal-associated pathogens *P. aeruginosa*, *H. pylori* and *C. jejuni*, which extensively glycosylate their flagella. In these species, flagellar glycosylation is absolutely essential for the biogenesis of functional flagella and hence for virulence^11^,^45^,^46^,^47^). To verify the modified sites identified by BEMAP, we used an orthogonal experimental setup similar to the methods applied to obtain the current knowledge about Flagellin glycosylation^46^,^47^,^48^,^49^. In this direct approach, an isolated Flagellin protein fraction was separated by SDS PAGE. The FliC protein was in-gel digested using trypsin and the resulting peptides were HILIC fractionated before analysis by nanoLC-MS/MS^50^. The acquired data were searched with either heptose or GlcNAc as variable modification. This experimental workflow identified 14 heptosylated Ser and Thr residues (Fig. 5A and Supplementary Table S6). A total of 12 of these sites were also found in the BEMAP analysis. The relatively low number of observed sites when applying an orthogonal experimental approach highlights the advantage of the BEMAP strategy. Our data also indicate that heptosylation is one of the most abundant sugar modifications in ETEC as no GlcNAc sites were identified on the most extensively modified protein FliC. To further characterize the FliC O-glycosylation, we mapped the modified FliC residues onto the primary sequence (see Fig. 5B). The majority of the O-glycosylation sites could be assigned to the N- and C-terminal domains, which are conserved amongst bacteria^51^. Interestingly, a sequence alignment of MG1655 and H10407 flagellin showed that the majority of the modified residues in the pathogen were conserved, but unmodified in the non-pathogen (see Fig. 5B). We mapped the O-glycosylation sites onto the *Salmonella* FliC crystal structure (Fig. 5C). This analysis illustrated that the sugar modifications were positioned on the interior face of the flagellum. The ETEC modification distribution is different from that of *C. jejuni* and *H. pylori* FliC, which are previously proposed to be glycosylated exclusively in the variable surface exposed domain^46^,^47^. In light of the close evolutionary relationship between the strains, a BEMAP analysis could be used to explore whether or not O-glycosylation is conserved feature in these pathogens. The glycosylated adhesin EtpA is mounted on the tip of the flagellum and interacts with the conserved domains of FliC^45^. It is plausible that the EtpA-FliC protein interactions could be augmented by glycosylation. Considering that a single flagellum may consist of as many as 30000 flagellin subunits the very large number of modifications make this extracellular appendage an substantial metabolic investment even if only a fraction of the sites are O-glycosylated^52^.

In addition, the combined dataset showed that ETEC Colonization Factor Antigen 1 (CFA/I) is O-glycosylated at two residues. Such modifications have been found to play an important role for the pathogenesis of other mucosa-associated pathogens^53^,^54^. Our analysis shows that the amino acid residues T74 and T78 are glycosylated. When mapping the glycosylated sites onto the CfaB crystal structure the glycans were found in the interface between CfaB subunits in the asymmetric unit (Supplementary Fig. S7). It is conceivable that the hydrophilic environment created by the glycans could facilitate fimbrial assembly.

### Searching for an ETEC O-linked protein glycosylation sequence motifs

**Figure 6:**
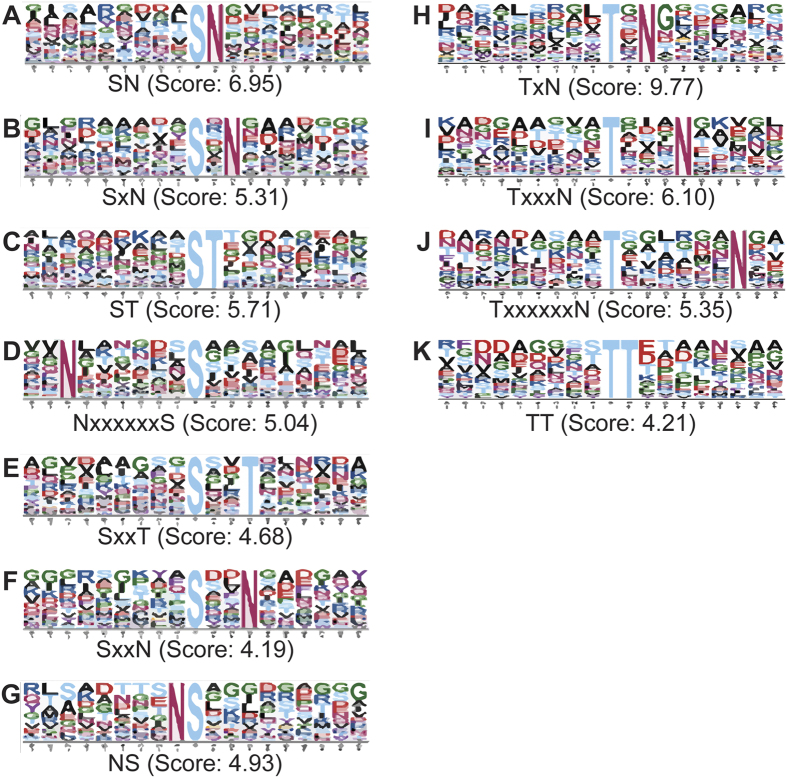
Definition of ETEC protein glycosylation sequence motifs. Glycosylated ETEC S/T residues identified in proteins associated with the outer membrane and outer membrane vesicles were centered with nine flanking residues using an in house developed script, see methods and materials. The motif-x algorithm^72^ was used to extract overrepresented patterns among 389 Serine and 239 Threonine sequences (*p value* < 0.0001). Figure (**A**–**G**) shows probable Serine sequence logos whereas (**H–K**) depicts Theronine motifs. A schematic motif sequence is listed below each panel as well as the probability score within each dataset.

The scarcity of identified *E. coli* O-linked glycosylation sites has led to the assumption that residue selectivity may rely on recognition through a structural arrangement spanning 19 amino acids rather than a linear sequence motif^32^,^55^. The obtained outer membrane glycosylation dataset allowed us to search for a sequence motif using bioinformatics prediction. Thus, we explored the nature of O-linked glycosylation in *E.coli* using the MotifX algorithm^56^. We searched for overrepresented motifs in a nine amino acid sequence window surrounding 389 and 239 unique Ser and Thr sites, respectively. An ETEC H10407 database was used as background and only motifs with high significance (*p* < 10^−4^) were considered. Seven serine and four threonine glycosylation motifs were significantly enriched in our data set comprising 37% of all identified sites (Fig. 6). In four out of five serine sequence outputs, glycosylation correlated with preference for asparagine in position −7, +1, +2 and +6 relative to the central character whereas the fifth motif contained a threonine in position +1. The threonine motifs were identical to the serine motifs. The glycosylation motif analysis extracted an overrepresentation of threonine in position +1 and asparagine in +2. Protein glycosylation in ETEC is proposed to be catalysed by the predicted *N*-acetylglucosamine (GlcNAc) transferase EtpC and the heptosyltransferase TibC which attaches heptoses to its target^16^,^39^. The unambiguous enrichment of asparagine flanking both the serine and threonine suggests that this amino acid is functionally important for glycosylation. It remains to be determined which glycosyltransferase recognises the individual motifs.

## Conclusion

We set out to devise a new method for accelerating the discovery of novel O-linked glycosylated proteins in *E. coli*. Our method, termed BEMAP, represents a refinement of a previous β-elimination/Michael addition experimental strategy originally described for phosphorylated peptides and O-GlcNAcylated peptides^26^. The novelty of BEMAP lies in the use of 2-Aminoethyl phosphonic acid (AEP) for nucleophilic peptide tagging. The selectivity of BEMAP is achieved by the glycan-to-phosphonate molecule exchange combined with a highly specific enrichment protocol for enrichment of the phosphonate peptide^28^. Importantly, the BEMAP chemistry can be applied in principle to any organism on a large-scale proteomics level irrespective of the chemical properties and nature of the O-linked sugar moiety. As demonstrated in Fig. 1, BEMAP replaces the carbohydrate moiety of a synthetic glycosylated peptide with a phosphonate tag in a chemical reaction exceeding 95% efficiency. We have shown that phosphorylated serine and threonine residues can be converted by the BEMAP chemistry, as previously published by Oda *et al*.^26^, and hence represent a source of false identification, see Fig. S2A,B. This can however be mitigated by including a simple Alkaline Phosphatase treatment of the sample prior to the BEMAP workflow (Fig. 3) or by including proper controls of enriched phosphopeptides from the sample.

Another limitation of BEMAP relates to establishing the identity of the eliminated sugar modification. Building on prior knowledge, ETEC modifies proteins with heptose and potentially GlcNAc monosaccharides^15^,^16^. However, when working with organisms for which the nature of O-glycosylation is limited or unknown, different approaches can be considered. In one workflow, monosaccharides from a purified protein can be identified using a direct chemical analysis^15^,^57^. Alternatively, the O-glycans can be released from individual proteins or whole cells, isolated and finally identified using advanced nanoLC-ESI-MS/MS^58^.

We highlight that BEMAP is compatible with experimental strategies which investigate the relative abundance of proteins such as stable isotope labeling with amino acids in cell culture (SILAC) and isobaric tags for relative and absolute quantitation (iTRAQ)^59^,^60^. We propose that BEMAP can improve our understanding of the intricacies of bacterial protein glycosylation amassed during pathogenesis which should lead to new opportunities to manipulate these pathways. In this regard, it should be noted that BEMAP constitutes a powerful qualitative glycoprotein discovery tool; no information about the modification frequency of a given amino acid residue is provided. It is highly conceivable that bacterial pathogens may exploit variable multisite glycosylation patterns to scramble their surface structure in order to evade recognition by the immune system. Indeed, a recent study^61^ demonstrated extensive antigenic variation in *Neisseria meningitidis* type IV pili. We propose the use of top-down mass spectrometric analysis subsequent to BEMAP glycosite discovery in order to determine the frequency of modification at specific sites of selected glycoproteins.

To identify specific pathogenic *E. coli* associated glycoproteins we compared the outer membrane protein complement to non-pathogenic reference strain MG1655 sampled under identical conditions. By applying our BEMAP workflow we identified a total of 618 glycosylated residues which could be assigned to 149 proteins, see Supplementary Table S5. By categorizing the identified ETEC outer membrane associated glycoproteins according to their gene ontology (GO) annotation^40^, our data indicate that protein glycosylation in *E. coli* plays a role in ETEC virulence as well as normal cellular physiology, see Figs 4 and 5 and Supplementary Fig. S6. Our findings parallel recently published data revealing an abundance of post translational modifications (PTMs) in *E. coli* including tyrosine phosphorylation, lysine succinylation and lysine acetylation as well as glycosylation in e.g. *H. pylori and A. baumannii*^24^,^25^,^34^,^62^,^63^,^64^. Using the motifX algorithm, our findings reinforce the notion that O-glycosylation residue selection in ETEC may rely on a sequence motif (Fig. 6). Previous studies have demonstrated that the *E. coli* heptosyltransferases are rather promiscuous in target selection^13^,^14^ and our data cannot discriminate whether EtpC and TibC compete for monosaccharide accepting Ser or Thr residues and whether they are capable of accepting both heptose and GlcNAc as substrate.

The presented work has exposed a significant amount of previously unexplored glycoproteins in *E. coli* that warrant deeper characterization. Our data supersedes any reported number of glycoproteins originating from a single microorganism^23^.

### Experimental Procedures

#### Strains

Pathogenic *E. coli* ETEC H10407 and *E. coli* K-12 MG1655 were used in this study. Bacterial strains are listed in Table 1.

### General methods

Cells were grown in M9 minimal medium containing 0.4% glucose^65^. Cultures were inoculated at an OD_600_ of 0.01 from an overnight (O/N) culture.

#### Purification of outer membrane associated proteins

Outer membrane proteins were isolated from cells grown to OD_600_ = 0.6 in 1.5 L M9 minimal medium supplemented with 0.4% glucose. Bacteria were harvested at 4500 × g for 30 min at 4 °C. The cell pellet was resuspended in 15 ml lysis buffer (50 mM sodium phosphate pH 7.0, 150 mM NaCl supplemented with EDTA-free protease inhibitor tablet from Roche) and lysed three times in a French press at 10,000 psi. Outer membrane associated proteins were obtained essentially as described by^37^. Outer membrane associated proteins were precipitated using the improved Wessel/Flügge method for large sample volumes^66^. Briefly described, the 20 ml outer membrane extract was vortexed together with 15 ml ice cold Methanol. 5 ml ice cold Chloroform was added and the sample was vortexed once again before centrifuged at 10000 × g for 45 min at 4 °C. The aqueous phase was carefully removed and 16.66 ml ice cold Methanol was added to the remaining protein sheet floating on top of the organic phase. The sample was vortexed and the protein precipitated by centrifugation at 10000 × g for 45 min at 4 °C. Organic solvent was removed and the protein pellet was allowed to air dry.

### Purification of membrane vesicles

Membrane vesicles were isolated from a cell culture grown to OD_600_ = 0.6 in 1.5 L M9 minimal medium supplemented with 0.4% glucose. A cell free culture supernatant was obtained by pelleting bacteria at 4500 × g for 30 min at 4 °C and sterile filtration (Millipore 0.22 μm filter cup). Supernatant was concentrated 21 fold using a stirred ultrafiltration cell model 8400 (Millipore) fitted with a PLCC ultrafiltration disc with a 5 kDa NMWL cut-off (Millipore). Vesicles were obtained from concentrated culture supernatant by centrifugation at 125000 × g for 3 hours at 4 °C. Proteins were isolated from the collected vesicles using the improved Wessel/Flügge method described above, by keeping the ratios between aqueous and organic solvents fixed.

### Outer membrane and MV in solution reduction, alkylation and proteolytic digestion

Outer membrane as well as MV associated protein samples were solubilized in 100 μl buffer containing 6M Urea, 2M thiourea, 100mM TEA bicarbonate pH 8.0 before reduced in 10 mm DTT for 1 hour at 25 °C and alkylated in 50 mM iodoacetamide for 40 minutes at 25 °C in the dark. Each sample was diluted 1:10 with 50 mm TEAB pH 8.0 and digested with 2% (w/w) trypsin 16 h at 25 °C. The supernatant was desalted using Oasis HLB Plus short cartridges (Waters) as recommended by manufacturer and finally dried by vacuum centrifugation and stored at −20 °C.

### Fetuin Alkaline Phosphatase treatment analysis

1nmol Fetuin was reduced in 10 mM DTT for 1 hour at 25 °C and alkylated in 50 mm iodoacetamide for 40 minutes at 25 °C in the dark in total reaction volume of 50 μl containing 10 mM TEAB pH 8.0. Fetuin sample was digested with 2% (w/w) trypsin 16 h at 25 °C before micro-tip desalted^67^ and finally dried by vacuum centrifugation and stored at −20 °C. Phosphopeptides were isolated using TiO_2_ enrichment protocol as described in ref. 28 and finally lyophilized. When required, Alkaline Phosphatase treatment was performed as recommended by manufacturer (Thermo Scientific. FastAP Thermosensitive Alkaline Phosphatase; EF0654). Samples were micro-tip desalted as described above before analysed by MALDI TOF MS (UltraFlex II, Bruker Daltonics, Bremen).

### Isolation of Flagellum for orthogonal ESI MS/MS verification

Flagellum was isolated from H10407 as described by^68^ with a few modifications. Briefly described, 200 ml of bacteria were grown into mid-exponential phase. One hundred OD_600_ units were harvested at 6000 × g for 15 min at 4 °C. The cell pellet was resuspended in 2 ml of 10 mM Tris-HCl pH 8, 75 mM NaCl and incubated at 60 °C for 20 min. A PRO200 homogenizer (PRO Scientific) set in position 2 (~10000 r.p.m.) was used to detach the fimbriae in two pulses lasting two minutes at 4 °C. A cell free supernatant was obtained by centrifugation at 14000 × g for 10 min at 4 °C and sterile filtration (0.22 μm). Flagellae and other proteins in supernatant were isolated using the improved Wessel/Flügge method as described above, by keeping the ratios between aqueous and organic solvents fixed.

### 1D SDS-PAGE and In-gel digestion

Crude FliC extract was re-suspended in 1x SDS loading buffer (60 mM Tris-HCl pH 6.8, 2% SDS, 10% glycerol, 0.005% bromophenol blue, 5 mM EDTA, 0.1 M DTT) to a final conc. of 0.5 OD_600_ unit/μl. Protein fractions were boiled for 5 min. Crude FliC fraction was separated on a 4–12% NuPage novex Bis-Tris mini gel (Invitrogen). FliC protein band was visualized using colloidal coomassie blue staining and subsequently excised^69^. Protein bands were in-gel digested^70^ and micro-tip desalted^67^ and finally dried by vacuum centrifugation and stored at −20 °C.

### BEMAP

BEMAP protocol was carried out with either 1.5 mg outer membrane or 0.3 mg vesicle derived peptides as input. Lyophilized peptide sample was resuspended in 25 μl Alkaline Phosphatase (AP) solution containing 10U of thermosensitive Alkaline Phosphatase (ThermoFischer scientific; EF0654). Phosphatase reaction continued for 45min at 37 °C. 75 μl BEMAP reaction mixture was added to the AP solution. The final concentration of BEMAP chemicals in the 100 μl reaction volume was 0.4 M 2-AEP (Sigma; 268674), 0.75 M NaOH (Sigma; S8045), 20 mM Ba(OH)_2_ (Sigma; 433373). The BEMAP reaction was incubate at 37 °C in a heating block for 3.15 hours shaking at 1300 r.p.m. The reaction was stopped by acidification (1% TFA final concentration). Sample volume was increased to 1 ml and the peptides were purified on an Oasis HLB Plus short cartridge (Waters) as recommend by manufacturer and subsequently lyophilized. TiO_2_ enrichment was performed as described by^28^.

### Mass spectrometric analysis of BEMAP samples

The BEMAP-enriched peptides were dissolved in 0.1% formic acid and separated by nano-LC-MS/MS on an in-house packed 17 cm × 100 μm Reprosil-Pur C18-AQ column (3 μm; Dr. Maisch GmbH, Germany) using an Easy-LC nano-HPLC (Thermo Scientific, Germany). The HPLC gradient was 0–34% solvent B (A = 0.1% formic acid; B = 90% ACN, 0.1% formic acid) in 180 mins at a flow of 250 nL/min. Mass spectrometric analysis was performed using an LTQ Orbitrap Velos (Thermo Scientific, Bremen, Germany). An MS scan (400–2000 *m*/z) was recorded in the Orbitrap at a resolution of 30,000 at 400 *m*/*z* for a target of 1e^6^ ions. The top seven most intense ions were fragmented by HCD MS/MS using the following parameters: activation time = 0.1 ms, normalized energy = 48, dynamic exclusion enabled with repeat count 1, exclusion duration = 30 s, intensity threshold = 5000, target ions = 2e^5^.

### HILIC fractionation

Tryptic peptides isolated from flagellin were resuspended in 90% ACN, 0.1% TFA and injected onto an in-house packed TSKgel Amide-80 HILIC (Tosoh, 5 μm) 320 μm × 170 mm μHPLC column using an Agilent 1200 HPLC system^50^. The peptides were eluted using a gradient from 90% ACN, 0.1% TFA to 60% ACN, 0.1% TFA over 35 mins at a flow rate of 6 μl/min. Fractions were automatically collected at 1 min intervals after UV detection at 210 nm and the fractions were combined to a total of 12–14 fractions according to UV detection. All fractions were dried by vacuum centrifugation.

### Orthogonal mass spectrometric analysis of FliC

Peptides were HILIC fractionated as described above before analyzed by an Easy-nLC and nanospray source (Thermo Fisher Scientific) coupled with a Q-Exactive Plus mass spectrometer (Thermo Fisher Scientific, Bremen, Germany). Approximately 1 μg of in-gel digested FliC peptide (5 μl) was reconstituted in 0.1% formic acid and loaded onto a trap column at 250 bar (2 cm length, 100 μm inner diameter, ReproSil, C18 AQ 5 μm 120 Å pore (Dr. Maisch, Ammerbuch, Germany)) vented to waste via a micro-tee and eluted across a fritless analytical in-house packed resolving column (17 cm length, 75 μm inner diameter, ReproSil, C18 AQ 3 μm 120 Å pore) with a 107 min gradient of 0–30% LC-MS buffer B (LC-MS buffer A: 0.1% formic acid; LC-MS buffer B: 0.1% formic acid, 95% ACN) using a flow rate of 300 nL/min. Instrument method consisted of one survey scan (AGC target value: 1e6; R = 70K; maximum ion time: 120 milliseconds; mass range: 400 to 1400 m/z, followed by data-dependent tandem mass spectra on the top 12 most abundant precursor ions ((isolation width: 1.6 m/z; HCD collision energy (NCE): 32; MS1 signal threshold: 2e4; AGC MS2 target value: 1e6; maximum MS/MS ion time: 200 milliseconds; dynamic exclusion: repeat count of 1, maximum exclusion list size, 20 seconds wide in time, +/−10 ppm wide in m/z; doubly-charged precursors only; Minimum signal threshold of 10,000.

### Analysis of Mass Spectrometry Data

Raw data generated on the LTQ Orbitrap Velos or Q-Exactive Plus mass spectrometer (Thermo Fisher Scientific, Bremen, Germany) were processed with Proteome Discoverer (Version 1.4.1.14, Thermo Fisher Scientific) and subjected to database searching using an in-house Mascot server (Version 2.2.04, Matrix Science Ltd., London, UK). Database searches were performed with the following parameters: Database: annotated *E. coli* proteomes from ETEC H10407, AIEC LF82, NMEC IHE3034 and commensal MG1655; Trypsin as the enzyme allowing a maximum of one missed cleavages sites; Carbomidomethylation of Cys as fixed modification; Deamidation of Asn and Gln; Oxidation of Met allowed as variable modification; When required BEMAP mass tag 2-AEP ((C(2) H(6) N O(2) P) on Ser/Thr or Heptose on Ser/Thr or GlcNAc on Ser/Thr was set as variable modification. Precursor and fragment mass tolerance were set to 10 ppm and 0.05 Da, respectively. Precursor mass range set from 350 Da to 7,000 Da. False discovery rate was set to 1% at peptide level using the Percolator algorithm.

Our in house developed tool Peptide Finder (accessible at www.microbiology.sdu.dk) was used to extract and compile a list of uniquely modified peptides using a ProteomeDiscover output text file as template. Similarly, our in house developed script MotifX aligner (accessible at www.microbiology.sdu.dk) was used to extract and centre Ser or Thr modified residues with nine flanking residues; Number of flanking amino acids is user defined. ProteinCenter (Thermo Scientific, Germany) was used to assign Gene Ontology terms to all identified proteins.

## Additional Information

**How to cite this article**: Boysen, A. *et al*. A novel mass spectrometric strategy ‘‘BEMAP’’ reveals extensive O-linked protein glycosylation in enterotoxigenic *Escherichia coli*. *Sci. Rep.* **6**, 32016; doi: 10.1038/srep32016 (2016).

## Supplementary Material

Tables S1-S6

Figure S1-S7

## Figures and Tables

**Table 1:** Strains used in this study.

Name	Genotype	Source/Reference
***E. coli***		
*MG1655*	Wild type K-12 strain	ATCC number 700926
**ETEC**		
H10407	Wild type (serotype 078:H11)	Gift from Statens Serum Institute
