# Supplementary material for: A novel mass spectrometric strategy “BEMAP” reveals Extensive O-linked protein glycosylation in Enterotoxigenic Escherichia coli
Source: Sci Rep. 2016 Aug 26;6:32016. doi: 10.1038/srep32016 (PMC5000012; doi:10.1038/srep32016)
Supplement: Figure S1-S7 [file srep32016-s2.pdf]

Supplementary Information

A novel mass spectrometric strategy "BEMAP" reveals Extensive O-linked protein glycosylation in Enterotoxigenic *Escherichia coli*

Anders Boysen, Giuseppe Palmisano, Thøger Jensen Krogh, Iain G. Duggin, Martin R. Larsen, Jakob Møller-Jensen

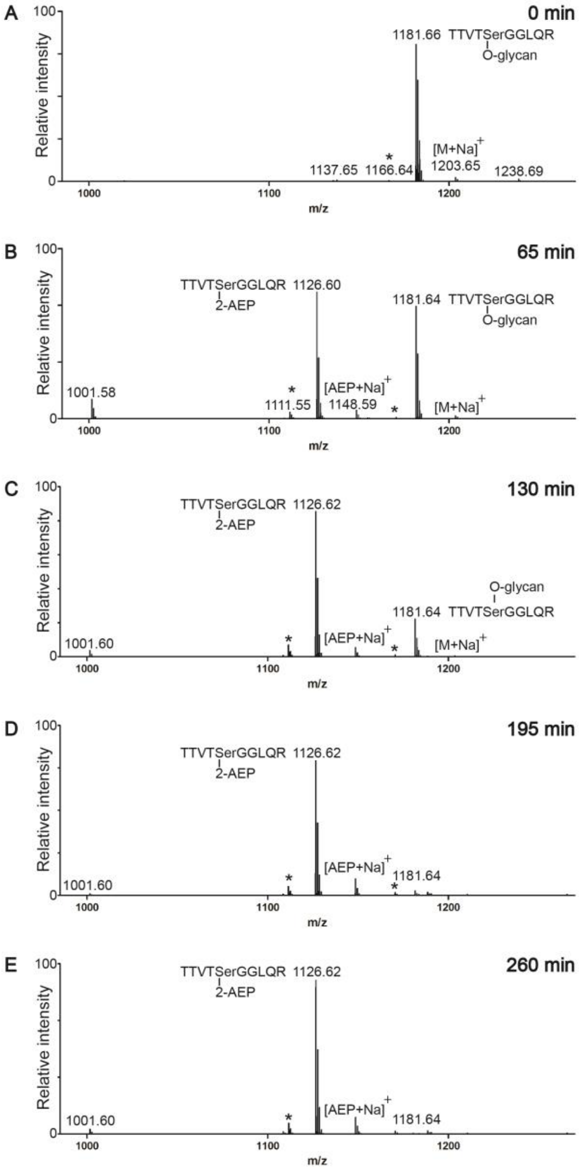

Figure S1: BEMAP convert glycopeptides into phosphopeptides in a time dependent manner. (A) MALDI MS spectrum of TTVTSGGLQR (m/z = 1181.59 Da) synthetic O-linked glycopeptide. Minor amounts of intact mass with sodium salt as well as a M-15 Da ion is observed prior to BEMAP reaction. (B-E) The BEMAP reaction replaces the carbohydrate moiety with the 2-AEP molecule and produces a phosphopeptide with the mass of 1126.64 Da. Minor traces of β-eliminated as well as intact peptide can be observed (m/z = 1001.62 Da and 1181.59, receptively) after 195 min.

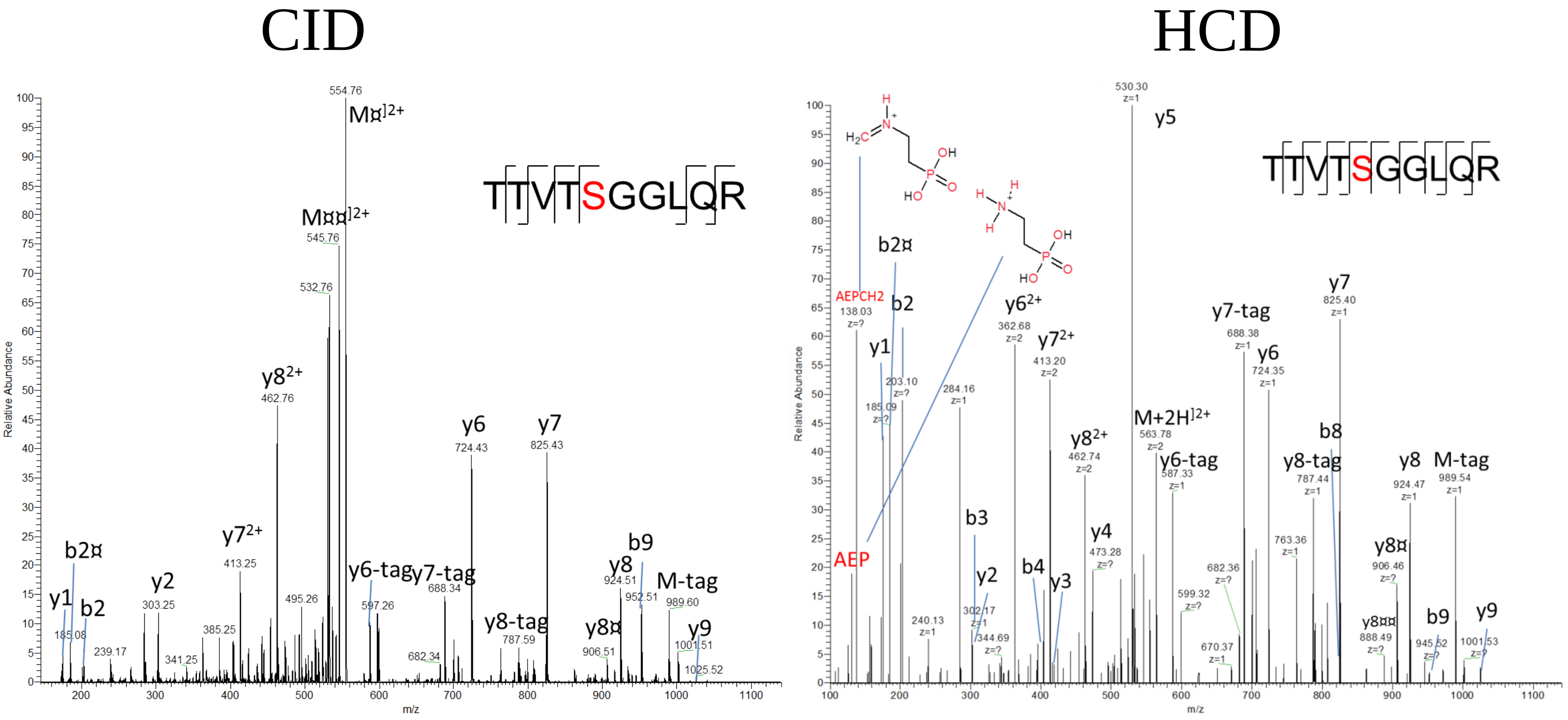

Figure S2: Gas-phase fragmentation behavior of 2-AEP moiety. The AEP addition greatly improves mapping of O-glycosylated amino acid residues by higher-energy collisional dissociation (HCD) fragmentation. Moreover, the AEP group yielded two characteristic reporter ions during HCD fragmentation (m/z=126.03 Da and m/z=138.03 Da), which are very useful for their identification and validation in complex MS/MS spectra.

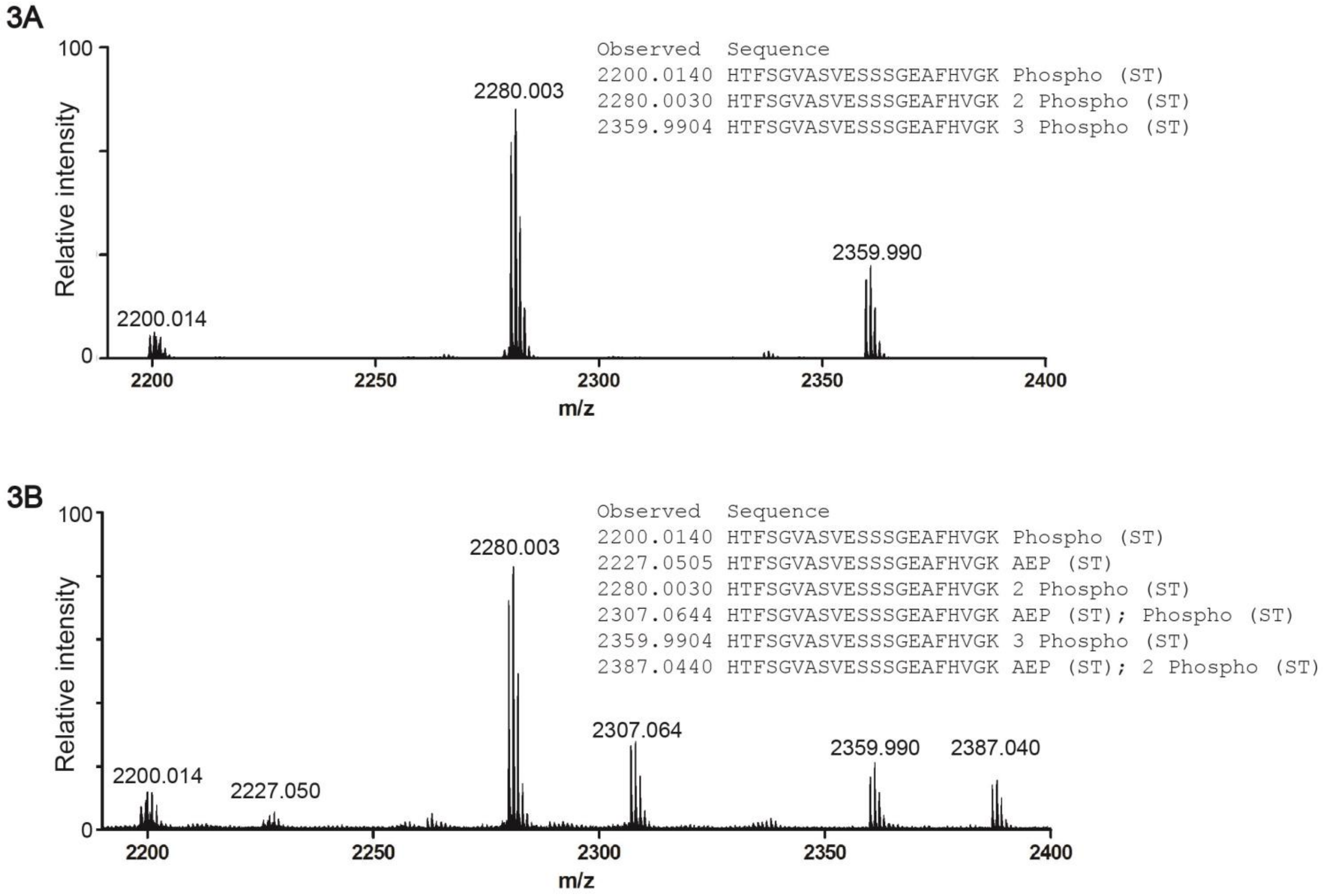

Figure S3: BEMAP partially convert phosphopeptides into 2-AEP tagged peptides. (A) In-solution digested Fetuin phosphopeptides were isolated using TiO_2 enrichment and then analysed by MALDI MS. (B) BEMAP treatment of phosphopeptides results in a modest conversion into the 2-AEP tag.

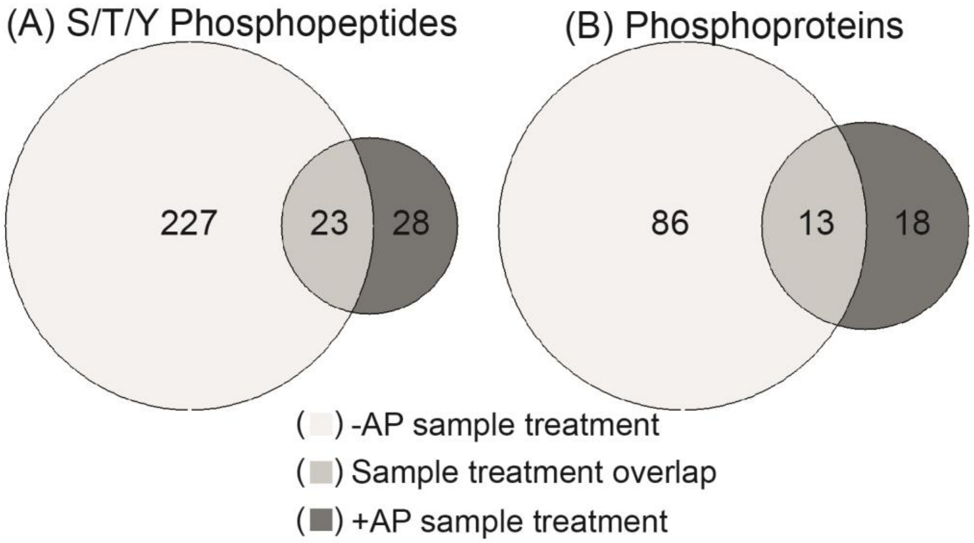

Figure S4: Outer membrane associated proteins are S/T/Y phosphorylated. Outer membrane proteins associated were sequentially isolated and digested with trypsin. The peptides were treated either with or without alkaline phosphatase (AP) prior to phosphopeptide enrichment and LC-MS/MS analysis. When comparing the two datasets, 23 phosphorylation sites were shared between 13 proteins. At the protein level, AP sample treatment significantly reduces the number of identified phosphoproteins.

5A: ATSVNsGGR

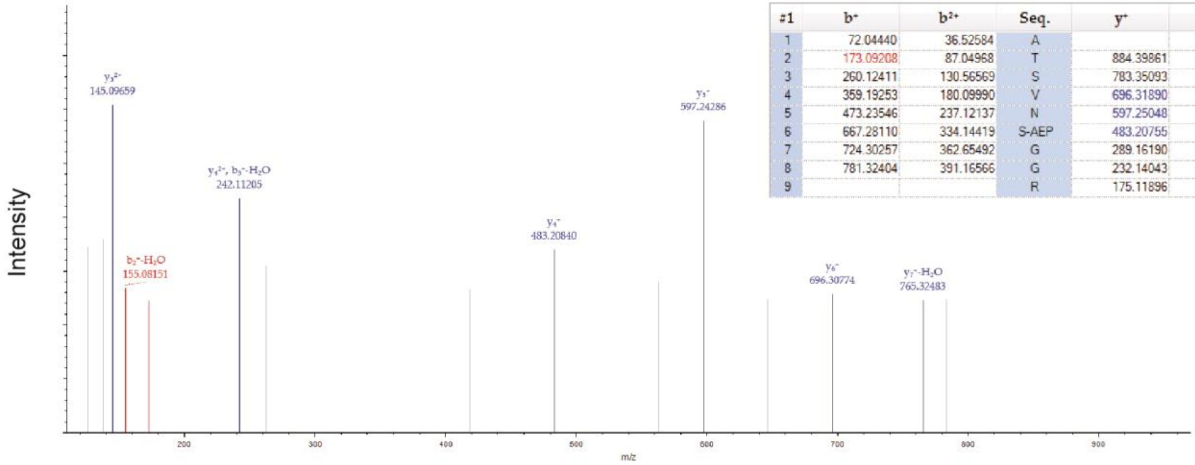

#1	b^+	b^{2+}	Seq.	y^+	y^{2+}	#2
1	72.04440	36.52584	A			9
2	173.09208	87.04968	T	884.39861	442.70294	8
3	260.12411	130.56569	S	783.35093	392.17910	7
4	359.19253	180.09990	V	696.31890	348.66309	6
5	473.23546	237.12137	N	597.25048	299.12888	5
6	667.28110	334.14419	S-AEP	483.20755	242.10741	4
7	724.30257	362.65492	G	289.16190	145.08459	3
8	781.32404	391.16566	G	232.14043	116.57385	2
9			R	175.11896	88.06312	1

5B: NTTINsGGK

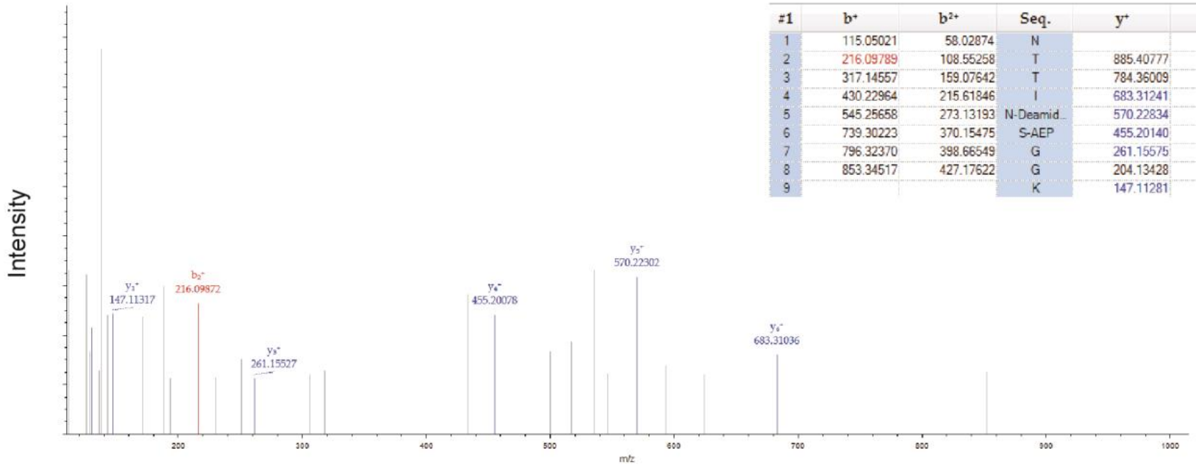

#1	b^+	b^{2+}	Seq.	y^+	y^{2+}	#2
1	115.05021	58.02874	N			9
2	216.09789	108.55258	T	885.40777	443.20752	8
3	317.14557	159.07642	T	784.36009	392.68368	7
4	430.22964	215.61846	I	683.31241	342.15984	6
5	545.25658	273.13193	N-Deamid...	570.22834	285.61781	5
6	739.30223	370.15475	S-AEP	455.20140	228.10434	4
7	796.32370	398.66549	G	261.15575	131.08151	3
8	853.34517	427.17622	G	204.13428	102.57078	2
9			K	147.11281	74.06004	1

Figure S5: Annotated MS/MS spectra showing two 2-AEP modified peptides which can be assigned to the TibA adhesin.

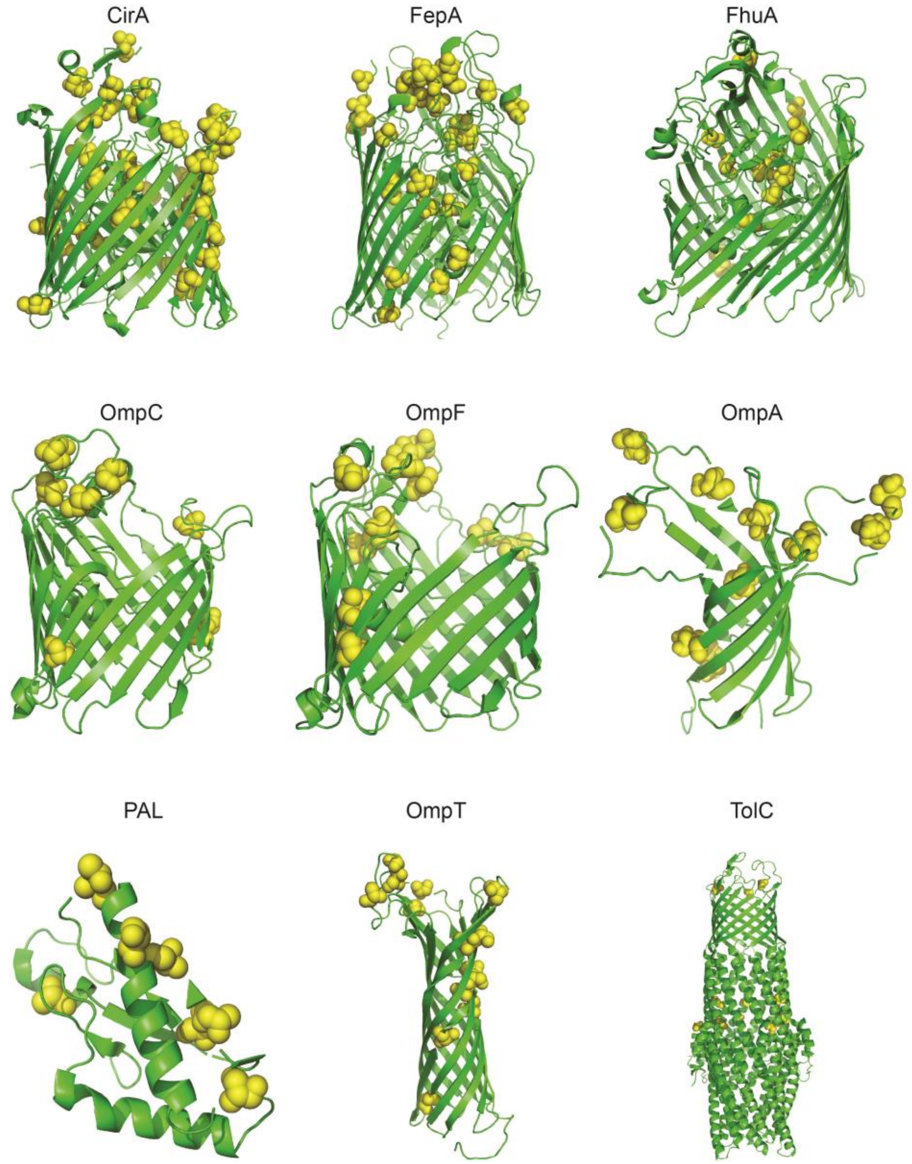

Figure S6: Mapping of ETEC outer membrane transporter protein glycosylation sites. The visualization of ETEC protein glycosylation was accomplished using crystalized *E. coli* K12 outer membrane transporters as template. Glycosylated residues identified in ETEC are shown in yellow spheres. The modified crystal structures of CirA, FepA, FhuA, OmpC, OmpF, OmpA, Peptidoglycan-associated lipoprotein, OmpT, TolC are adapted from Abergel et al., 2001; Basle et al., 2006; Buchanan et al., 1999; Buchanan et al., 2007; Cierpicki et al., 2006; Cowan et al., 1995; Ferguson et al., 2000; Vandeputte-Rutten et al., 2001.

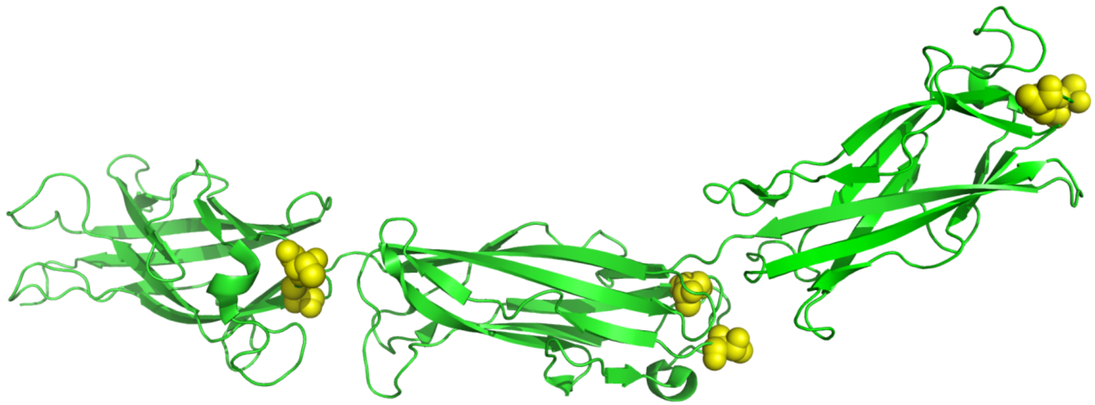

Figure S7: Mapping of ETEC H10407 CfaB, Colonization factor antigen subunit B, glycosylation sites. The three monomers are shown from a “top view” angel. Each monomer docks in an “end to end” fashion. The yellow spheres indicate O-glycosylated residues. Modified crystal structure is adapted from Li, Y. F., Poole, S., Nishio, K., Jang, K., Rasulova, F., McVeigh, A., Savarino, et al. PNAS, 2009.

Reference List

1. Abergel, C., Walburger, A., Chenivesse, S., and Lazdunski, C. (2001). Crystallization and preliminary crystallographic study of the peptidoglycan-associated lipoprotein from Escherichia coli. Acta Crystallogr. D. Biol. Crystallogr. 57, 317-319.

2. Basle, A., Rummel, G., Storici, P., Rosenbusch, J.P., and Schirmer, T. (2006). Crystal structure of osmoporin OmpC from E. coli at 2.0 A. J Mol. Biol. 362, 933-942.

3. Buchanan, S.K., Lukacik, P., Grizot, S., Ghirlando, R., Ali, M.M., Barnard, T.J., Jakes, K.S., Kienker, P.K., and Esser, L. (2007). Structure of colicin I receptor bound to the R-domain of colicin Ia: implications for protein import. EMBO J 26, 2594-2604.

4. Buchanan, S.K., Smith, B.S., Venkatramani, L., Xia, D., Esser, L., Palnitkar, M., Chakraborty, R., van der Helm, D., and Deisenhofer, J. (1999). Crystal structure of the outer membrane active transporter FepA from Escherichia coli. Nat. Struct. Biol. 6, 56-63.

5. Cierpicki, T., Liang, B., Tamm, L.K., and Bushweller, J.H. (2006). Increasing the accuracy of solution NMR structures of membrane proteins by application of residual dipolar couplings. High-resolution structure of outer membrane protein A. J Am. Chem. Soc. 128, 6947-6951.

6. Cowan, S.W., Garavito, R.M., Jansonius, J.N., Jenkins, J.A., Karlsson, R., Konig, N., Pai, E.F., Pauptit, R.A., Rizkallah, P.J., Rosenbusch, J.P., and . (1995). The structure of OmpF porin in a tetragonal crystal form. Structure. 3, 1041-1050.

7. Ferguson, A.D., Welte, W., Hofmann, E., Lindner, B., Holst, O., Coulton, J.W., and Diederichs, K. (2000). A conserved structural motif for lipopolysaccharide recognition by procaryotic and eucaryotic proteins. Structure. 8, 585-592.

8. Vandeputte-Rutten, L., Kramer, R.A., Kroon, J., Dekker, N., Egmond, M.R., and Gros, P. (2001). Crystal structure of the outer membrane protease OmpT from Escherichia coli suggests a novel catalytic site. EMBO J 20, 5033-5039.
